# Prevalence and seasonal pattern of enteric viruses among hospitalized children with acute gastroenteritis in Samawah, Iraq

**DOI:** 10.25122/jml-2021-0158

**Published:** 2022-01

**Authors:** Hazim Talib Thwiny, Nawar Jasim Alsalih, Zeayd Fadhil Saeed, Ali Mosa Rashid Al-Yasari, Moyed Abd AlHussein Al-Saadawe, Mohenned Abd ElHussein Alsaadawi

**Affiliations:** 1. College of Veterinary Medicine, University of Basrah, Basrah, Iraq; 2. Division of Microbiology, College of Veterinary Medicine, Al-Muthanna University, Samawah, Iraq; 3. Nursing Department, Al Furat Al Awsat Technical University, Samawah, Iraq; 4. Division of Physiology, College of Veterinary Medicine, Al-Muthanna University, Samawah, Iraq; 5. Central South University, Changsha, China; 6. Department of Parasitology, College of Veterinary Medicine, Al-Muthanna University, Samawah, Iraq

**Keywords:** viral diarrhea, astroviruses infections, adenoviruses infections, rotavirus infections, Samawah

## Abstract

Almost all of the deaths happening under the age of 5 occur in the developed countries of Africa and Asia. This study included children admitted to the surgical care, aged 6 months to 5 years, who suffered from acute gastroenteritis and received treatment at Samawah, Iraq, from December 2018 to December 2019. Test results detected different types of rotaviruses, adenoviruses, astroviruses using ELISA. 56.6% of the infections were attributed to a viral pathogen. The main cause was attributed to rotavirus and adenovirus. The causative agents of diarrheal diseases in 28.1% of cases are rotaviruses, in 17.05% – adenoviruses, in 11.43% – astroviruses. Viral mono-infections are detected more often than mixed infections. Viral intestinal infections are characterized by seasonality and rise in the cold season, with a peak incidence of rotavirus infection in April, adenovirus infection in November, and astrovirus infection in December.

## Introduction

Rotavirus infection is responsible for more than 500,000 deaths annually in children smaller than 5 years of age worldwide, with the majority of these deaths occurring in the developing countries of Africa and Asia [1]. There is a clear change in the etiological significance of pathogens that cause acute diarrheal diseases in children [2]. The common cause of acute intestinal infections in children is induced by viruses [3]. According to the World Health Organization (WHO), almost every child, regardless of socioeconomic status, suffers from viral diarrhea during the first 5 years of life, causing a great impact on the health system [4]. It is well known that young children are the most vulnerable group for viral diarrhea [5]. The results of large studies indicate significant geographical differences in the prevalence of viral diarrhea.

It should be noted that the prevalence of rotavirus, adenovirus, and astrovirus infections in children and the features of their seasonal distribution remain insufficiently studied [5]. However, the study of the etiological structure of viral diarrhea is of great practical importance for determining measures of prevention, monitoring, and treatment [6]. Nevertheless, research indicates that the main possible causes of viral diarrhea are related to rotavirus, followed by astrovirus and adenovirus, which were noticed to be more spread in the cold season [7]. This study aimed to investigate the prevalence of rotaviruses, adenoviruses, and astroviruses as acute viral intestinal infections in children, depending on the season of the year.

## Material and Methods

The study population involved children from 6 months to 5 years who suffered from acute gastroenteritis and were hospitalized in the Gynecology and Children Hospital, Samawah, Iraq, from December 2018 until December 2019. All patients were examined for general clinical, biochemical, bacteriological examinations of feces to detect *Shigella*, *Salmonella*, and conditionally pathogenic microflora.

Clinical samples were collected during the first days of illness but no later than the third day from the onset of the disease. Patients’ feces were collected in disposable plastic containers with a transport medium containing preservative materials with highly hygienic precautions. The clinical samples were frozen to -20°C until the time of the study. To allow re-analysis, the material was stored at -70°C. The collected feces samples were tested by ELISA for the presence of rotaviruses, adenoviruses, astroviruses using R-Biopharm RIDASCREEN, Germany test system. Chrome agar was used to diagnose *Shigella* and *Salmonella* species.

Patients were selected randomly throughout the seasons of the year. A prerequisite for the examination of patients was the collection of samples for bacteriological culture and ELISA on the first day of admission to the hospital before the start of therapy. Diagnostic criteria for viral diarrhea in children were anamnestic data: symptoms of acute onset of gastroenteritis, short incubation period, family case history or cases in the children’s groups, seasonality, and laboratory results which were represented as the absence of bacterial pathogens in the feces of patients and the detection of antigen (virus) through ELISA.

Statistical analysis of the data was carried out following generally accepted methods of variation statistics using Graph Pad Prism version 8. Differences were considered statistically significant at p<0.05.

## Results

**Figure 1.**
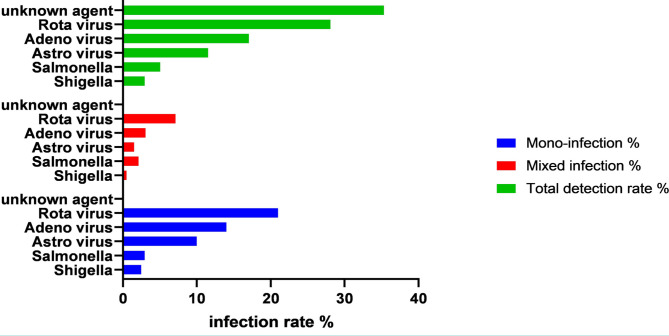
The frequency detection of mono- and mixed infections in patients with acute intestinal infections.

Pathogens-related acute intestinal diarrhea during the study course was recorded in 64.67% (679/1050) of diarrheic children. Among the recorded cases, rotaviruses were the most often detected in 295/1050 (28.1%) patients, adenoviruses in 17.1% (179/1050), astroviruses in 11.5% (121/1050) (Figure 1). Out of 1050 samples with diarrheal diseases, the viral antigens were detected in 595 samples, at a rate of 56.7% (595/1050). The results indicated a high prevalence of viral diarrhea in Samawah. Bacterial infections were detected only in 84/1050 (8.0%) of patients, significantly less than the rate of rotaviruses, adenoviruses, astroviruses infections, and unknown-cause diarrhea. Two kinds of bacteria were detected: *Salmonella* and *Shigella*, at rates of 5.1% (54/1050) and 2.9% (30/1050), respectively (Figure 1). In the other cases, 35.33% (371/1050) of total diarrheic cases have no clear causes of diarrhea and are diagnosed as unknown cause (Figure 1).

More than half, 50.7% (532/1050) of cases were due to mono-infection, while the mixed infection was 14% (147/1050). The mono infections mainly related to viral causes (significant differences from bacterial agents) as the positive results for rotavirus, adenovirus, and astrovirus were 21% (220/1050), 14% (147/1050), and 10% (105/1050), respectively which represented 45% (472/1050) of total diarrheic cases (Figure 1). However, the bacterial agents were individually detected in 5.43% (54) patients distributed as 2.9% (30/1050) due to *Salmonella* and 2.3% (24/1050) due to *Shigella* (Figure 1). The prominent viral infection was caused by rotaviruses which were significantly higher than other viruses.

The frequency of mono viral diarrhea over the entire observation period over different seasons can change throughout the year. In the winter-spring period (January–May), there was the highest prevalence of rotavirus infection, then the number of adenovirus infections increased from September to February (autumn-winter). Astroviruses were diagnosed in one-year children, significantly increasing from October to December. The seasonal appearance of rotavirus was clear as the incidence increased in winter. However, there is a further increase in the spring and some sporadic cases in summer. The maximum values were in March and April, and the peak incidence was observed in April. The lowest frequency of detection of rotavirus infection in children was recorded from June to October. Adenovirus infection was also characterized by the seasonality of incidence: the elevated incidence was observed from September to February, with a maximum peak in November. The number of detected positive samples of astroviruses varied throughout the year. In September, the proportion of detected astroviruses was 0.95% (10/1050). The increased incidence of astroviral diarrhea occurred in October and November; the peak incidence was in December 26.7% (280/1050), and finally, the most prevalent infections were rotavirus and adenovirus.

**Figure 2.**
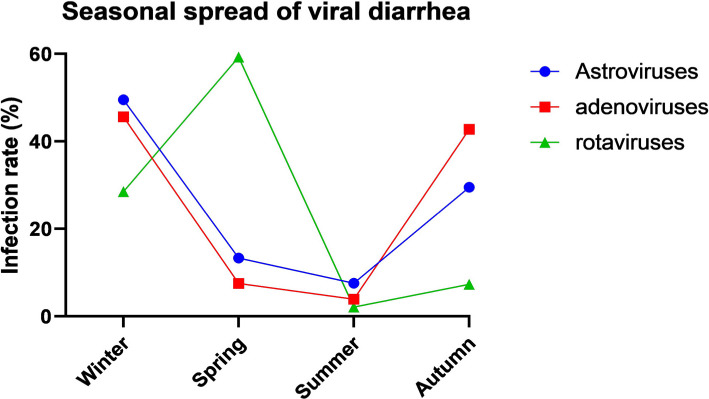
The seasonal spread of viral diarrhea.

In winter, among the mono-infections, astroviruses prevailed, which were detected in 49.5% of cases, followed by adenoviruses (45.6%), and finally rotaviruses (28.5%). In the spring, rotaviruses dominated (59.3%), followed by astroviruses (13.3%) and adenoviruses (7.5%). Astrovirus infection was more often detected in children (7.6%) during the summer. Finally, adenovirus infection was 42.8% in the autumn with a maximum frequency rate in December, while rotaviruses and astroviruses were found in 7.3% and 29.5% of cases, respectively (Figure 2).

The recorded samples of viral diarrhea in children were differently spread according to seasons. These differences were significantly different. The data was analyzed using One sample T-test using GraphPad Prism 8.

## Discussion

Acute gastroenteritis in children under age 5 is still one of the most common illnesses in infants and toddlers. In developed countries, this is a prominent cause of hospitalization, and in underdeveloped nations, it is a major source of childhood morbidity and mortality [8]. Three major enteric viruses have been identified as the most important etiological agents of acute gastroenteritis in children around the world, notably group A rotavirus, adenovirus, and astrovirus [9, 10].

This study included 1050 children aged 6 months to 5 years old admitted to the Gynecology and Children Hospital in Samawah, Iraq, with acute gastroenteritis. Adenovirus types 40 and 41 and group A rotaviruses, astroviruses, and adenoviruses. From December 2018 to December 2019, our inquiry used ELISA techniques to analyze stool samples for viral agents that cause diarrhea. Previous research has suggested that ELISA, which is easier to use, faster, and less expensive than RT-PCR, may be sensitive enough for routine diagnostic work [11]. PCR can be used to genotype and analyze the homogeneity of viral strains in circulation across years and geographies. In Samawah children, we found a significant incidence of enteric viruses, with 56.7 percent of stool samples containing at least one enteric virus. In Europe, 35 to 40% prevalence rates have been reported, with 43.6% in India and 53.7% in Africa [12–14].

In the current study, the most prevalent virus was group A rotavirus, linked to 28.1% of infants and children under five, indicating that rotavirus infection is the leading cause of gastroenteritis. This finding was in line with the findings of other previous research conducted worldwide. Rotaviruses are the most common cause of gastroenteritis in children, accounting for 15% to 40% of all occurrences of diarrhea [15–20]. As a result, programs aimed at reducing acute gastroenteritis in infants and young children should prioritize the prevention of group A rotavirus infection. The creation of immunization and vaccination programs for children at high risk is the only known adequate primary preventive intervention against rotavirus illness. Human adenovirus was the most commonly detected virus, with a frequency of 17.1%. Human adenovirus has been found in gastroenteritis investigations in poor and industrialized countries, with prevalence rates ranging from 2 to 35% [21–25].

The detection rates in the current study are significantly higher than previously published research on human adenovirus in infants with diarrhea from other Middle Eastern countries such as Kuwait (4%) [26], Qatar (6.25%) [27], and Saudi Arabia (8%) [28] but similar to Egypt (20%) [29]. The causes for the increased discovery of human adenovirus are unknown. The prevalence of astroviral infections in this study was 11.5%, comparable to other studies such as 14% in Brazil [30] and 13.3% in Spain [31]. Other research (e.g., Japan, Greece, Iran, and India) have found that this pathogen circulates less than other enteric viruses [32–35]. The incidence of these viruses is most likely tied to each country’s geographical features and socioeconomic situations [23, 36–39].

Co-infections with adenovirus, astrovirus, rotavirus A, and enteropathogenic bacteria were found in 14% of the cases studied in this investigation. Other investigations found co-infections at rates ranging from 2.1 to 50% [40–43]. Similar findings have previously been reported in China [11] and France [21], where 9% and 17% of dual infections, respectively, have been documented. Dual infections raise the question of whether a single virus causes sickness or whether two viruses work together to potentiate one another. The underlying cause of the diarrheal sickness is unknown, although earlier studies found no statistically significant difference in clinical symptoms between mono infections and dual infections [11, 21, 44].

Seasonal variations in the prevalence of the gastroenteritis virus were detected in this investigation (Rotavirus, Adenovirus, and Astrovirus). Rotavirus showed high seasonality in children under five throughout the monitoring period, with a majority in the winter-spring period (January–May). Rotavirus gastroenteritis was at its peak in Europe in late winter or early spring [45–48]. Observational studies of human rotavirus sickness have revealed that low temperatures, low humidity, and low precipitation levels are linked to an elevated risk of rotavirus infection and may produce ideal circumstances for Rotavirus spread, transmission, and maintenance in the environment [49, 50]. Indeed, cold weather drives people to congregate in more enclosed spaces, exposing sensitive persons to surfaces or items that have been polluted more frequently and intensely [51]. Although greater frequencies were recorded from September to February, human adenovirus was discovered in most of the months for which data was collected (autumn-winter).

The human adenovirus discovered in Greece was not shown to have any seasonal pattern, according to Levidiotou *et al.* [33]. Human adenovirus was found in the fall and winter months in Turkey, according to Ozdemir *et al.* [52]. As previously reported from other countries [18, 53–55], the largest prevalence of astrovirus infections was shown to spread from October to December (the fall and winter). However, there are indications that the prevalence of astrovirus is higher in the spring and summer months than in winter [36, 56, 57]. The reason for the seasonal differences in astrovirus patterns is unknown. One drawback of the current study is that the true viral infection prevalence may be higher than predicted. We only examined hospitalized children with moderate to severe gastroenteritis, and the proportion of viral etiological agents among children who only received home care or outpatient visits was not estimated. The current study did not explore caliciviruses, group C rotaviruses, sapoviruses, and toroviruses, and more research is needed to fully understand the etiology of viral diarrhea in Iraqi children.

To test the constancy of this seasonal trend, more research with a longer time span and bigger areas in Iraq is needed. According to this study, viral diarrhea is one of the major etiological causes of diarrheal illnesses in children. Rotaviruses and adenoviruses are the primary causes. Therefore, it can be said that viral diarrhea is the major type of diarrhetic case. Viral intestinal infections are seasonal and increase during the cold season, with rotavirus infection peaking in April, adenovirus infection peaking in November, and astrovirus infection peaking in December.

## Acknowledgments

### Conflict of interest

The author declares no conflict of interest.

### Ethical approval

The study was approved by the Ethical Committee of Veterinary Medicine School at Al-Muthanna University, no. 122018.

### Personal thanks

The authors acknowledge the staff of Microbiology Laboratory of the Gynecology and Children Hospital, Samawah, Iraq, who help collect the samples. Many thanks to the Microbiology Department of Veterinary Medicine School staff at Al-Muthanna University, who facilitated confirming the microbiological tests.

### Authorship

MAA and AMRAY contributed to the conception and design of the study, interpretation of data and final approval of the version to be submitted. HTT and NJA contributed to acquisition of data and drafting the article, preparing, and reading the histopathology slides. ZVS contributed to collecting the samples and designing the study and methodology. MAASA contributed to collecting the samples and designing the study and methodology.
